# *Poria cocos* Lanostane Triterpenoids Extract Promotes Collagen and Hyaluronic Acid Production in D-Galactose-Induced Aging Rats

**DOI:** 10.3390/life13112130

**Published:** 2023-10-28

**Authors:** Chien-Liang Chao, Han-Peng Kuo, Hsin-Wen Huang, Maw-Yeun Cheng, Hsin-Fan Chao, Shih-Min Lu, Hang-Ching Lin, Chao-Jih Wang, Tsu-Chung Chang, Chi-Rei Wu

**Affiliations:** 1 Sinphar Pharmaceutical Co., Ltd., Sinphar Group, Yilan 269, Taiwan; chaokmc@sinphar.com.tw (C.-L.C.); hwhuang@sinphar.com.tw (H.-W.H.); swyu@sinphar.com.tw (M.-Y.C.); hfchao@sinphar.com.tw (H.-F.C.); smlu@sinphar.com.tw (S.-M.L.); lhc@sinphar.com.tw (H.-C.L.); 2 SynCore Biotechnology Co., Ltd., Sinphar Group, Yilan 269, Taiwan; hpkuo1@syncorebio.com; 3 School of Pharmacy, National Defense Medical Center, Taipei 114, Taiwan; 4 Sinphar Tian-Li Pharmaceutical Co., Ltd., Sinphar Group, Hangzhou 311100, China; zrwang@sinphar.com.tw; 5 Department of Biochemistry, National Defense Medical Center, Taipei 114, Taiwan; 6 Department of Chinese Pharmaceutical Sciences and Chinese Medicine Resources, China Medical University, Taichung 404, Taiwan

**Keywords:** *Poria cocos*, aging, lanostane triterpenoids, Lipucan^®^, collagen, hyaluronic acid

## Abstract

The global aging population is expanding at an increasingly rapid pace, with approximately one-fourth of the world’s population expected to be composed of elderly individuals by 2050. Aging skin is one of the major characteristics expressed in the elderly. The study comprehensively utilizes both cell and animal experiments to confirm the skin anti-aging effects of *Poria cocos* (*P. cocos)*, which is one of the most important traditional Chinese medicines classified as tonic Chinese medicine, commonly used to treat physical weakness and aging-associated diseases. We demonstrate in this study that *P. cocos* lanostane triterpenoids extract (Lipucan^®^) ameliorates aging skin and promotes collagen accumulation and hyaluronic acid production in galactose-induced aging rats. Purified lanostane triterpenoids were initially identified as active components in *P. cocos*, which significantly increased collagen and hyaluronic acid levels in cultured human skin cells.

## 1. Introduction

Human skin has many physiological functions. The skin is the organ of the body with the largest surface area. Among these many functions, it has a basic role as the body’s first protective barrier to defend against bacteria, virus, chemicals, particulates, solar ultraviolet, and even mechanical injury [1,2,3,4,5]. Because of direct and frequent contact with the exterior environment, the skin usually suffers more damage than most other organs in the body. The skin consists of three main layers, the epidermis, dermis, and hypodermis [5]. The epidermis is the outermost skin layer. The epidermis outer layer is the stratum corneum, which consists of approximately 15–20 layers of keratinocytes and provides most of the effective barrier function [4,5,6,7]. The other epidermis layers are the stratum lucidum (only in the hands and feet), stratum granulosum, stratum spinosum, and stratum basale. The dermis layer has comprehensive elasticity, strength, and moisturizing functions, containing the nervous, lymphatic, and vascular systems and extracellular matrix proteins such as collagen and those for skin moisture such as hyaluronic acid [8]. Collagen is one of the main and most abundant proteins in the human extracellular matrix. It is found in the dermis, ligaments, tendons, vessel walls, and other connective tissues. Collagens are the most abundant proteins in mammals, and they occupy about 70–80% of the dry skin weight and contribute to the dermis structural integrity. The collagen family contains 28 types with one or more triple-helical domains. Among them, types I and III are the major types of collagens in the skin [9,10]. Hyaluronic acid (also known as Hyaluronan, HA) is a glycosaminoglycan with basic structure composed of repeating disaccharide units (D-glucuronic acid and N-acetylglucosamine) [8]. HA is most abundant in the human skin, making up almost 50% of the total body. HA plays a key role closely related to skin moisture because of the strong water retention capacity [8,11,12]. HA in the dermis regulates water balance, osmotic pressure, and ion flow; functions as a sieve to exclude certain molecules and strengthen the cell extracellular domain; and stabilizes skin structures using electrostatic interactions [8]. Skin health is always associated with skin intrinsic (natural) and extrinsic (environmental) aging such as ultraviolet exposure [13]. Both intrinsic and extrinsic aging cause damage to skin function and make a huge change in the skin’s appearance, such as in skin inelasticity, collapse, wrinkles, and dryness. Collagen and HA both play important roles in skin health. The production of collagen and HA continues to decline during human aging [8,13,14]. The elderly population has rapidly increased by nearly threefold from 1950 to 2010 and continues to increase. Skin care has always been one of the most important issues that people care about in the world [15]. Many studies demonstrated that certain natural products and herbal medicines can support collagen and hyaluronic acid production, suggesting their efficacy in fighting against skin aging [16]. For example, a good deal of research indicated that vitamin C (ascorbic acid) increases collagen synthesis, especially type I and type III [17,18]. *Camellia japonica* oil, *Panax ginseng*, cinnamon, and *Emblica officinalis* have all been reported to promote collagen production in human skin cells [19,20,21,22]. *Bacillus subtilis* natto-fermented Radix astragali enhanced hyaluronic acid production in human skin cells [23]. Despite these studies, there are still many unknown active compounds from natural products that need to be explored and understood to promote skin collagen and hyaluronic acid production.

The dried sclerotium of the saprophytic and edible fungus *P. cocos* (Schwein) F.A. Wolf (syn. *Wolfiporia cocos*) in the Polyporaceae family, called Fuling, is one of the most common and important traditional Chinese medicines, belonging to tonic medicine that is widely used in Eastern countries. It has been reported to display wide bioactive effects, including diuretic [24], sedative [25], anti-diabetic [26], immunostimulatory [27,28], anti-inflammatory [29,30], anti-tumor [31], and anti-bacterial [32] activities. The main active constituents of *P. cocos* are a group of lanostane triterpenoids, which are considered to contribute most of its pharmacological activities [33,34]. This study investigates and explores the potential activities of *P. cocos* to promote collagen and hyaluronic acid production and the anti-aging activity of *P. cocos* via in vitro and in vivo studies.

## 2. Materials and Methods

### 2.1. Poria cocos Extract Preparation (PCE) (Lipucan^®^)

**Figure 1:**
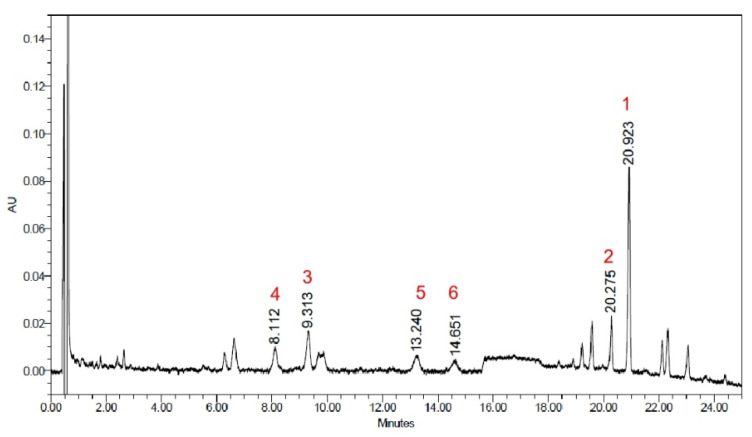
The UPLC chromatography of PCE.

**Figure 2:**
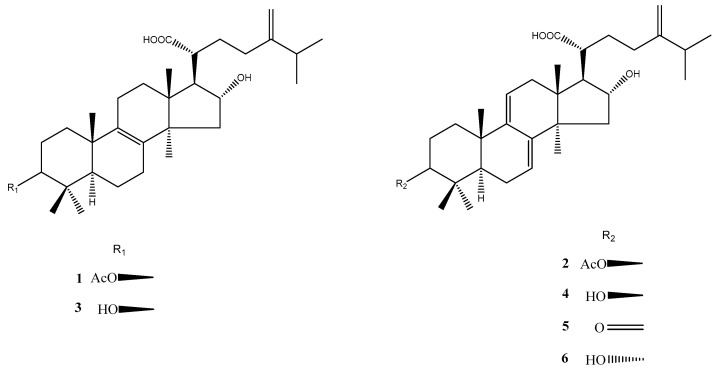
The chemical structures of lanostane triterpenoids **1**–**6** isolated from *P. cocos*. **1**: pachymic acid; **2**: dehydropachymic acid; **3**: tumulosic acid; **4**: dehydrotumulosic acid; **5**: polyporenic acid; **6**: 3-epi-dehydrotumulosic acid.

The dried sclerotium of *P. cocos* was extracted using 75% ethanol to obtain *P. cocos* extract (PCE) (Lipucan*^®^*), which was produced by Sinphar Tian-Li Pharmaceutical Co, Ltd., Sinphar Group, Hangzhou, China. The PCE was analyzed by ultra-high-performance liquid chromatography (UPLC, ThermoDionexUltiMate 3000 UHPLC system, Waltham, MA, USA) (Figure 1) and the total content of six lanostane triterpernoid compounds (Figure 2, compounds **1**~**6**) was 21.81%.

### 2.2. Isolation and Purification of Lanostane Triterpenoid Compounds *(**1**–**6**)*

*P. cocos* dried sclerotium (10 kg) was extracted three times by refluxing with 75% ethanol for 3 h. The concentrated extract was chromatographed on silica gel (70–230 mesh) using increasingly polar mixtures of CH_2_Cl_2_–MeOH (CH_2_Cl_2_: MeOH, 97:3; CH_2_Cl_2_: MeOH, 96:4; CH_2_Cl_2_: MeOH, 90:10; and MeOH only). According to the analytical thin-layer chromatography (TLC), four fractions (Fr.1~Fr.4) were collected for further separation. Fr.1~Fr.3 were subjected to HPLC preparation on a Waters XBridge RP-18 column (250 mm × 19 mm, 5 μm, Milford, MA, USA) using 85% methanol as the mobile phase system. The flow rate was 18 mL/min and six major peaks of interest were selectively collected. The fractions containing the targeted compounds were further condensed to dryness and produced pachymic acid (**1**) (106 mg), dehydropachymic acid (**2**) (53 mg), tumulosic acid (**3**) (120 mg), dehydrotumulosic acid (**4**) (68 mg), polyporenic acid C (**5**) (16 mg), and 3-epi-dehydrotumulosic acid (**6**) (12 mg). Their structures were elucidated using Nuclear Magnetic Resonance (NMR) spectroscopy (Table S1) and electrospray ionization mass (ESI-MS) analyses (Figures S2, S4, S6, S8, S10 and S12) and by comparison with the literature data [35] (Figure 2).

### 2.3. Animals

Male SD (Sprague Dawley) rats (200–250 g) were obtained from BioLASCO Co., Ltd., Taiwan. They were housed in groups of three, chosen at random, in wire-mesh cages (39 cm × 26 cm × 21 cm) in a temperature- (23 ± 1 °C) and humidity (60%)-regulated environment with a 12 h/12 h light/dark cycle (light phase: 08:00 to 20:00). The Institutional Animal Care and Use Committee of China Medical University approved the experimental protocol (CMUIACUC-2018-243). These animals were cared for according to the Guiding Principles for the Care and Use of Laboratory Animals. After a one-week acclimatization, the rats were randomly grouped according to different doses and used for experiments.

### 2.4. D-Galactose-Induced Rat Skin Aging Model

Thirty SD rats (10 weeks old) were used in this experiment, randomly divided into five groups of six rats each. The six rats of the normal group were subcutaneously injected (S.C.) with saline for 8 weeks and then orally given distilled water for 4 weeks. The remaining twenty-four rats were designated to the D-galactose-induced skin aging group. The induction procedure was modified using the previous literature [36]. Briefly, rats were treated with D-galactose (100 mg/kg/day, s.c.) in normal saline solution for 8 weeks. D-galactose-induced skin aging rats were then randomly divided into four groups of 6 rats each. The six rats of the D-galactose-induced skin aging group (control group) were orally treated with distilled water daily for 4 weeks after 8 weeks of D-galactose treatment. The remaining eighteen rats were designated to the PCE treatment groups. These groups were divided into PCE-L, PCE-M, and PCE-H groups, in which the rats were orally given either PCE-L (1 mg/kg), PCE-M (3 mg/kg), or PCE-H (6 mg/kg) after 8 weeks of D-galactose treatment. After 4 weeks of distilled water or PCE administration, all rats were sacrificed to collect skin tissues from the rat’s back. The rat’s left back skin tissues were used for hematoxylin and eosin (HE) or immunohistochemical (IHC) staining. The skin tissues from the rat’s right back were used for assaying type I collagen protein expression and the hyaluronic acid content.

### 2.5. Immunohistochemical (IHC) Staining Procedure

The rat left back skin tissues were cut into 1 cm^2^ pieces and fixed in 10% formalin. Post-fixation, paraffin slices were prepared and cut into sections (10 μm) using a microtome (Leica Biocut 2030, USA). The paraffin was removed from some skin sections, rehydrated using conventional histological techniques, and then stained with HE (Leica Biosystems, Nussloch GmbH-Nuβloch, Nußloch, Germany) for a morphological assessment. Some sections were incubated with a mouse anti-collagen I monoclonal antibody (Abcam plc; Cambridge, UK) at 4 °C. Subsequently, the histological sections were incubated with anti-mouse biotinylated secondary antibody and the immunohistochemistry reaction was amplified using an avidin–biotin–peroxidase reagent using a Vectastain Elite ABC kit (Vector Laboratories, Burlingame, CA, USA). Digital pictures were taken using 10× objectives (Nikon, Tokyo, Japan). Results were expressed as the average intensity of the positive immunoreactive cells of type I collagen immunohistochemistry (positive pixels)/the full area captured (total pixels) using the image processing and analysis software Java 7 (Windows version, National Institutes of Health, Bethesda, MD, USA).

### 2.6. Western Blot Analysis of Type I Collagen In Vivo

The skin tissue supernatants were subjected to Western blot analyses to determinate the type I collagen protein expression. The protein concentration was quantified using a Bradford protein assay kit (Bio-Rad, Hercules, CA, USA) followed by electrophoretic separation through SDS-PAGE. After transferring the protein samples to PVDF membranes, the samples were blocked with 5% non-fat dry milk and 0.1% tween-20 in tris-buffered saline at room temperature for 1 h. The membranes were then incubated with primary antibodies against type I collagen (Santa Cruz Biotechnology, Dallas, TX, USA) overnight at 4 °C and subsequently incubated with horseradish peroxidase-conjugated goat anti-rabbit or goat anti-mouse IgG. Signals were visualized by an enhanced chemiluminescence detection kit (Thermo Fisher Scientific Inc., Waltham, MA, USA) and a LAS-4000 mini-imaging system (Fujifilm, Kanagawa, Japan), and the optical density data were analyzed using MultiGauge v3.0 software (Fujifilm, Kanagawa, Japan). For the Western blot analyses, β-actin (Proteintech, Rosemont, IL, USA) served as an internal control.

### 2.7. Hyaluronic Acid Measurement In Vivo

The hyaluronic acid quantitative measurement in the rat’s right back skin was carried out in accordance with the manufacturer’s protocols [37]. Briefly, the skin tissues were washed with ice-cold PBS (10 μM, pH = 7.4) to remove excess hemolysis blood thoroughly and were then minced to small pieces. One gram of tissue was homogenized in 9 vol ice-cold PBS (containing a protease inhibitor solution (0.4 M NaCl, 0.05% Tween 20, 0.5% bovine serum albumin, 0.1 mM phenylmethylsulfonylfluoride, 0.1 mM benzethonium chloride, 10 mM EDTA, 10 μg/mL aprotinin)) with an ultrasonic cell disrupter on ice. The homogenates were then centrifuged at 12,000 rpm for 5 min at 4 °C to retrieve the supernatant. The supernatants were then analyzed for the hyaluronic acid content using an enzyme-linked immunosorbent assay (ELISA) kit (Bioscience Inc., San Diego, CA, USA).

### 2.8. Lanostane Triterpenoid Compounds *(**1**–**6**)* for Collagen and Hyaluronic Acid Analysis in Primary Human Dermal Fibroblasts

Primary human dermal fibroblasts (HDFs) (ATCC) were cultured in fibroblast basal medium supplemented with a serum-free fibroblast growth kit in a water-saturated atmosphere with 5% CO_2_ at 37 °C. The experiments were conducted 24 h after the cells had been seeded in 6-well (2 × 10^5^ cells/well) sterile clear-bottom plates. Various doses of lanostane triterpenoids (**1**–**6**) of *P. cocos* were dissolved in the culture medium and incubated for 48 h in HDF cells. The culture medium was then collected and concentrated. Equal amounts of proteins from each sample were analyzed for the collagen and hyaluronic acid levels. For the type I collagen level, Western blotting analysis was performed using primary antibodies against type I collagen (Santa Cruz, CA, USA) and β-actin (Arigo, Shanghai, China), and signals were detected using enhanced chemiluminescence kits (Amersham Biosciences, Little Chalfont, Buckinghamshire, UK). The hyaluronic acid production was assessed using an enzyme-linked immunosorbent assay (ELISA) kit according to the manufacturer’s protocol (Echelon Bioscience, Salt Lake, UT, USA).

### 2.9. Statistical Analysis

All data are shown as mean ± standard error of the mean (SEM). Statistically significant differences among groups were analyzed using one-way analysis of variance (ANOVA) (SigmaPlot 11.0 program). Different values between experimental and control groups were statistically significant at *p* < 0.05.

## 3. Results

### 3.1. The Anti-Aging Activity of PCE in D-Galatose-Induced Skin Aging Rats

**Figure 3:**
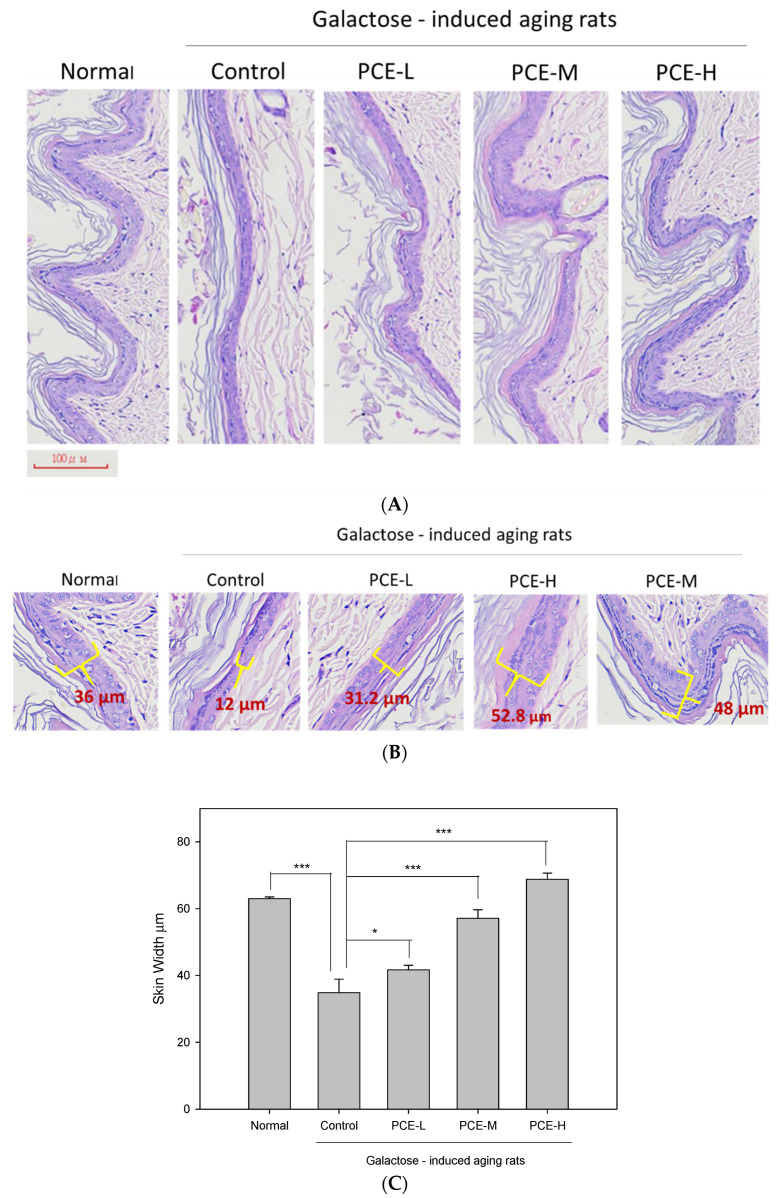
PCE-L (1 mg/kg), PCE-M (3 mg/kg), and PCE-H (6 mg/kg) effects on skin in D-galactose-induced skin aging rats: (**A**) skin papillary ridge; (**B**) skin epidermis thickness; (**C**) skin width. Columns indicate mean ± SEM (*n* = 6). * *p* < 0.05, *** *p* < 0.001 compared with control group.

The skin is characterized using a multi-layered structure consisting of the epidermis and dermis. Skin aging histological features include reduced epidermal thickness, flattening of the dermis and epidermis junction, also known as papillary ridge, and altered dermal structure [38]. The papillary ridges of D-galactose-induced skin aging rats (control group) became flattened (Figure 3A). PCE visibly improves skin flattening induced by D-galactose in a dose-dependent manner. The PCE-H papillary ridge apparently returned to its normal protruding appearance (Figure 3A). In addition, PCE protects the epidermis from thickness reduction due to skin aging (Figure 3B). After the total skin width measurement, PCE increased skin thickness in the D-galactose-induced skin aging group (Figure 3C).

### 3.2. PCE Promoted Collagen Type I Production in D-Galactose-Induced Skin Aging Rats

**Figure 4:**
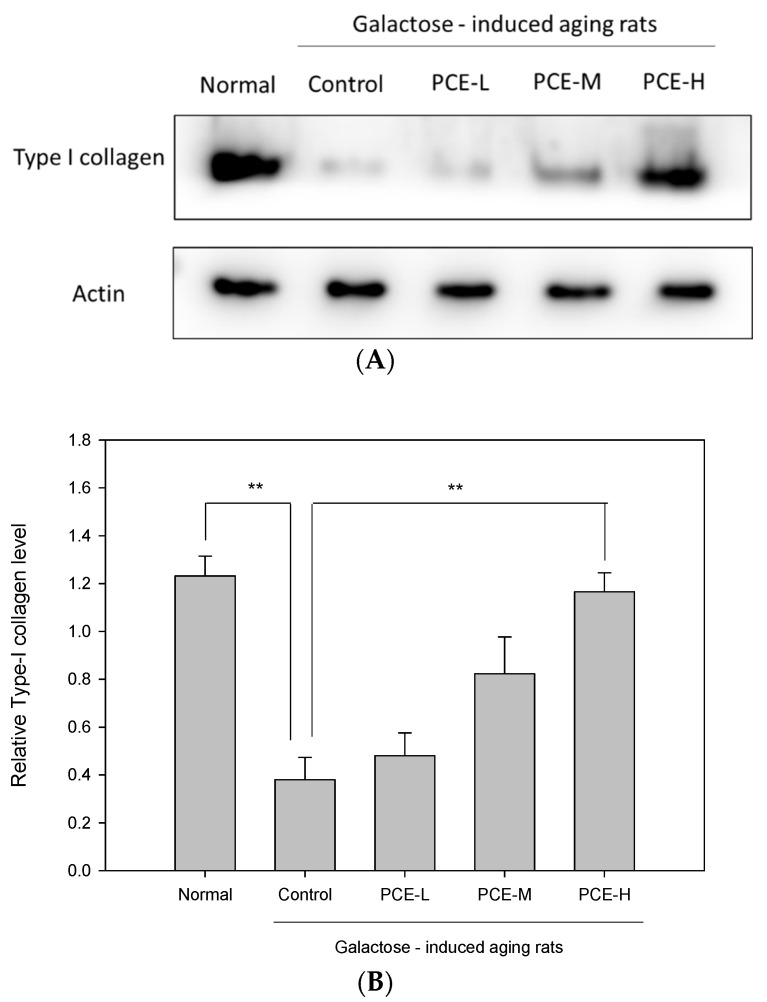
PCE-L (1 mg/kg), PCE-M (3 mg/kg), and PCE-H (6 mg/kg) effects on skin type I collagen in D-galactose-induced skin aging rats. (**A**) Western blotting analysis; (**B**) quantitative analysis shows that PCE-H significantly promoted type I collagen expression in D-galactose-induced skin aging rats. Columns indicate mean ± SEM (*n* = 6). ** *p* < 0.01 compared with control group.

To assess the PCE effect in rats with D-galactose-induced skin aging, the type I collagen protein expression in skin tissue was analyzed using Western blot analysis. The result shown in Figure 4 indicated that the type I collagen protein expression in galactose-induced skin aging rat skin tissue decreased to 69.12% compared with that in normal rat skin tissue (*p* < 0.01). We found that PCE-H significantly promoted type I collagen skin tissue protein expression in D-galactose-induced skin aging rats.

### 3.3. PCE Promoted Hyaluronic Acid Production in D-Galactose-Induced Skin Aging Rats

**Figure 5:**
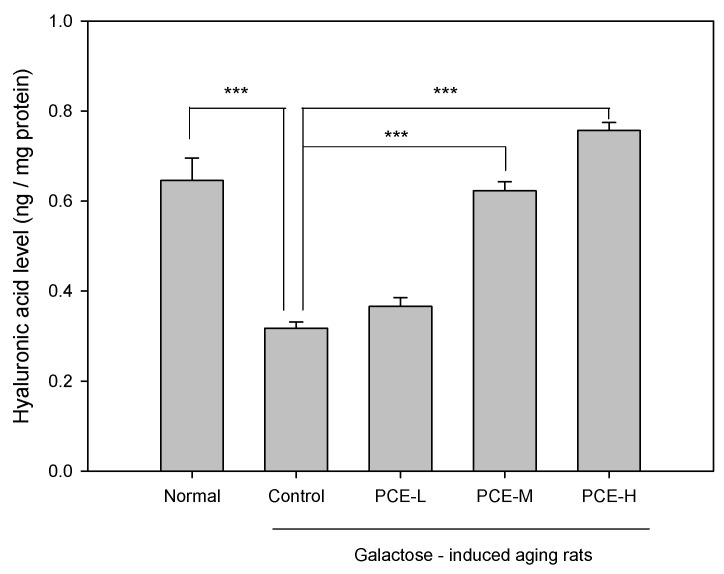
PCE-L (1 mg/kg), PCE-M (3 mg/kg), and PCE-H (6 mg/kg) effects on hyaluronic acid of skin tissue in D-galactose-induced skin aging rats using ELISA analysis. PCE-M and PCE-H significantly promoted hyaluronic acid production of skin tissue in D-galactose-induced aging rats. Columns indicate mean ± SEM (*n* = 6). *** *p* < 0.001 compared with control group.

To assess the PCE effect in D-galactose-induced skin aging rats, the hyaluronic acid level in skin tissue was also analyzed using ELISA analysis. The results shown in Figure 5 indicated that the skin tissue hyaluronic acid level in D-galactose-induced skin aging rats decreased to 50.84% compared with that of skin tissue in the normal rats (*p* < 0.001). We found that PCE-M and PCE-H both significantly promoted hyaluronic acid production of skin tissue in D-galactose-induced skin aging rats.

### 3.4. Isolation and Identification of Six Lanostane Triterpenoid Compounds ***1**–**6*** of P. cocos

Dried P. cocos (10 kg) was extracted three times by refluxing with 75% ethanol for 3 h. The concentrated extract was chromatographed on silica gel and a C18 column to furnish six major lanostane triterpenoid compounds: pachymic acid (**1**) (106 mg), dehydropachymic acid (**2**) (53 mg), tumulosic acid (**3**) (120 mg), dehydrotumulosic acid (**4**) (68 mg), polyporenic acid C (**5**) (16 mg), and 3-epi-dehydrotumulosic acid (**6**) (12 mg) (Figure 2). Their structures were elucidated using NMR spectroscopy (Table S1) and ESI-MS analysis (Figures S2, S4, S6, S8, S10 and S12) and comparison with the literature data [32,33]. The UPLC chromatograms of **1**–**6** are shown in Figures S1, S3, S5, S7, S9 and S11.

### 3.5. The Effects of Lanostane Triterpenoid Compounds ***1**–**6*** of P. cocos on Type I Collagen and Hyaluronic Acid Production in HDF Cells

**Figure 6:**
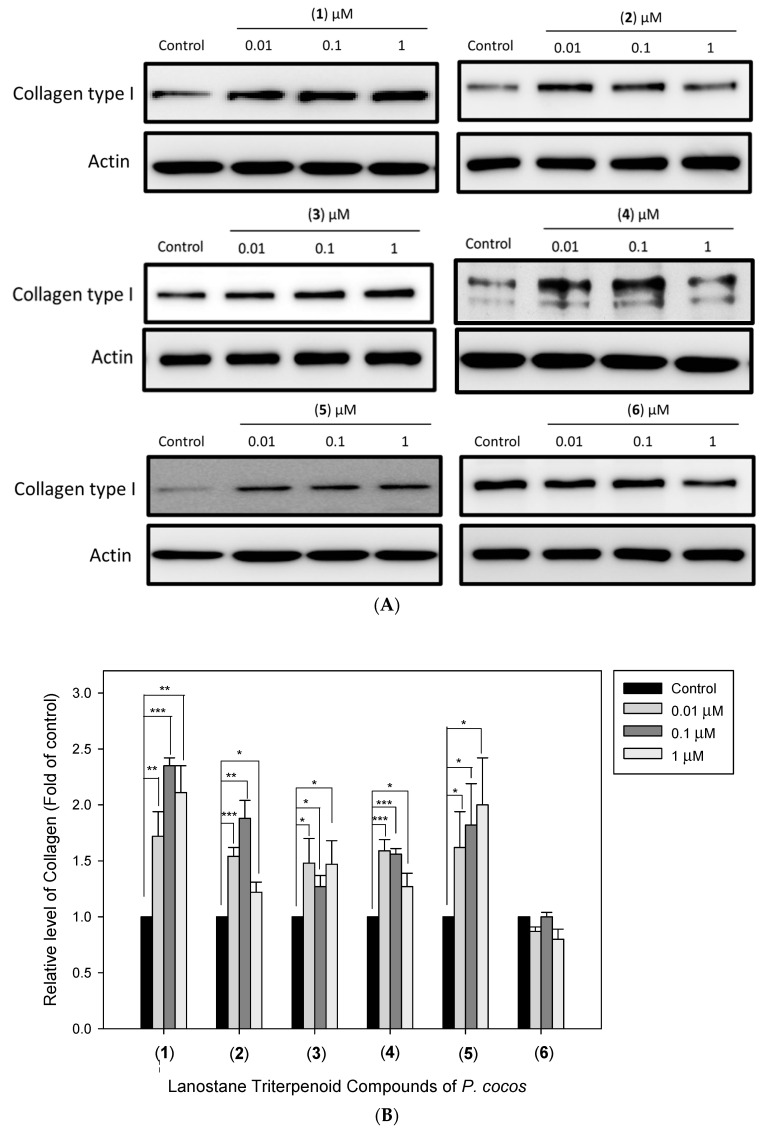
Effect of lanostane triterpenoid compounds (**1**–**6**) of *P. cocos* on the protein expression of type I collagen in HDF cells. The cells were incubated with or without the indicated concentrations of each compound for 24 h. (**A**) The relative expression level of type I collagen in HDF cells (**B**) quantified using the ImageJ Gel Analysis program (https://imagej.nih.gov/ij/download.html) The relative amount of type I collagen was calculated in HDF cells. Data are presented as the mean ± SD (*n* = 3; * *p* < 0.05, ** *p* < 0.01, *** *p* < 0.001).

**Figure 7:**
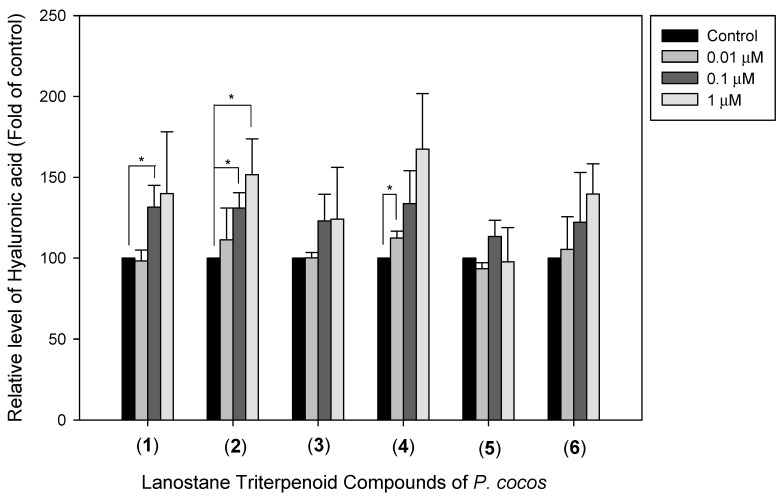
Effect of lanostane triterpenoid compounds (**1**–**6**) of *P. cocos* on hyaluronic acid level in HDF cells. The cells were incubated with or without the indicated concentrations of each compound for 24 h. The relative amount of hyaluronic acid produced in HDF cells. Data are presented as the mean ± SD (*n* = 3; * *p* < 0.05).

To identify the identified *P. cocos* lanostane triterpenoid compounds’ effects, we evaluated the effect of compounds **1**–**6** on the type I collagen protein expression and hyaluronic acid production in HDF cells. HDF cells were pre-incubated with the above-mentioned compounds for 48 h. From the results shown in Figure 6, 1 μM of lanostane triterpenoid compounds (**1**), (**2**), (**3**), (**4**), and (**5**) enhanced the expression of type I collagen (2.11 ± 0.24, 1.22 ± 0.09, 1.47 ± 0.21, 1.27 ± 0.21, and 2.00 ± 0.42-fold with respect to the control sample, with *p* < 0.01, *p* < 0.05, *p* < 0.05, *p* < 0.05, and *p* < 0.05, respectively). An amount of 0.1 μM of lanostane triterpenoid compounds (**1**), (**2**), (**3**), (**4**), and (**5**) enhanced the expression of type I collagen (2.35 ± 0.07, 1.88 ± 0.16, 1.27 ± 0.1, 1.56 ± 0.05, and 1.82 ± 0.37-fold with respect to the control sample, with *p* < 0.001, *p* < 0.01, *p* < 0.05, *p* < 0.001, and *p* < 0.05, respectively). An amount of 0.01 μM of lanostane triterpenoid compounds (**1**), (**2**), (**3**), and (**4**) enhanced the type I collagen expression (1.27 ± 0.22, 1.54 ± 0.08, 1.48 ± 0.22, and 1.59 ± 0.1-fold with respect to the control sample, with *p* < 0.01, *p* < 0.001, *p* < 0.05, and *p* < 0.001, respectively). These results indicated that these compounds significantly enhanced the type I collagen protein expression in HDF cells. In addition to its promoting effect on the type I collagen protein expression, the effects of compounds (**1**)–(**6**) on hyaluronic acid production were also assessed. The results shown in Figure 7 indicated a significant increase in hyaluronic acid in HDF cells at 1μM of (**2**), 0.1 μM of (**1**) and (**2**), and 0.01 μM of (**4**) by 151.69%, 131,53%, 131.05%, and 112.41% (*p* < 0.05, *p* < 0.05, *p* < 0.05, and *p* < 0.05, respectively) compared to the control cells.

## 4. Discussion

The prolongation of human life span around the world has led to a dramatic increase in the number of elderly people. By 2050, one-sixth of the world’s population will be over the age of 65 [39]. Aging is one of the most important issues in modern society as it can seriously affect the socio-economic and health cost burden. Anti-aging has always been an important research topic. Maintaining a healthy elderly population is an important issue that needs to be handled carefully. With the increase in age, the skin epidermis thickness increases and the collagen and hyaluronic acid contents in the dermis are significantly reduced. *P. cocos* has been traditionally used with a long history and was first recorded in “*Shen Nong’s Materia Medica*” [40]. It was classified as a tonic supplement and was believed to prolong human life. At present, there is little in-depth research on *P. cocos’s* anti-aging effects and the exploration of active compounds in *P. cocos.* Therefore, the anti-aging activities of *P. cocos* in skin were investigated and the active ingredients were elucidated in this study. The skin is the largest organ of the human body. It protects the human body from direct environmental influences [2]. It is the reason that skin health is so important for humans. People are willing to use healthy food or cosmetics with functional ingredients to improve skin aging and maintain a good skin condition. Physiologically, skin aging shows thinning of the epidermis, decreased collagen production in the dermis, and reduced skin elasticity. The decrease in hyaluronic acid reduces moisturizing ability and skin stretching, resulting in the loss of protective function and wound healing. Previous studies indicated that mice treated with D-galactose are generally considered to be an ideal animal model for studying oxidative damage and skin aging and *P. cocos* can effectively alleviate oxidative stress-related skin aging [41,42]. We showed in this study that PCE obviously recovered the skin tissue aging induced by D-galactose in a dose-dependent manner. Aging-induced flattening of papillary ridges in the dermis was induced by D-galactose but PCE restored the normal papillary ridge protrusion structure. In addition, PCE obviously increased skin epidermis and skin width in a dose-dependent manner. Furthermore, the protein expression of type I collagen and the hyaluronic acid in the skin tissues of rats were analyzed to clarify the basis of anti-aging by PCE. In this study, we proved that PCE remarkedly promoted the protein expression of type I collagen and the hyaluronic acid contents in the skin tissue of D-galactose-induced skin aging rats. These results suggest that PCE may increase type I collagen and hyaluronic acid to restore the papillary ridge wavy structure in D-galactose-induced aging rats. Previous reports indicated that lanostane triterpenoids were considered to be the major compounds in the ethyl acetate fraction based on HPLC analysis [28]. But, it remains unclear whether lanostane triterpenoids are active ingredients in skin anti-aging activity. We isolated, purified, and identified six lanostane triterpenoids from *P. cocos* and investigated their effects on type I collagen protein expression and the hyaluronic acid content in the HDF cell model. We showed that lanostane triterpenoid compounds (**1**–**4**) significantly enhanced the type I collagen expression in HDF cells. In addition, we also found that lanostane triterpenoid compounds (**1**), (**2**), and (**4**) significantly increased the hyaluronic acid level in HDF cells. Thus, these lanostane triterpenoid compounds might play a beneficial role in promoting the protein expression of type I collagen and the production of hyaluronic acid, which could be deeply related to strategies for ameliorating skin aging. In the future, further research will be conducted to explore the antioxidative mechanisms of *P. cocos* and its relationship with the promotion of collagen and hyaluronic acid production.

## 5. Conclusions

This study is the first to demonstrate the anti-aging action of PCE and its lanostane triterpenoid components on skin aging. PCE obviously recovered skin tissue aging induced by D-galactose in a dose-dependent manner. PCE promoted the protein expression of type I collagen and the production of hyaluronic acid to restore the dermis papillary ridge wavy structure in D-galactose-induced skin aging rats. Further evidence from cell experiments showed that the identified active PCE ingredients enhance type I collagen protein expression and hyaluronic acid production. Our findings support the lanostane triterpenoid components of *P. cocos* as promising candidates for development into improved therapies for skin aging. The antioxidant mechanism of *P. cocos* and its relationship with the promotion of collagen and hyaluronic acid production require further research. PCE and its proven active ingredients can be used to develop cosmetics or food supplements.

## Data Availability

The data presented in this study are available on request from the corresponding authors. The data are not publicly available.

## Supplementary Materials

The following supporting information can be downloaded at: https://www.mdpi.com/article/10.3390/life13112130/s1, Table S1. 1H NMR (400 MHz) and 13C (100 MHz) Spectroscopic Data for **1**–**6**. Figure S1. The UPLC chromatogram of pachymic acid (**1**). Figure S2. The ESI-MS spectrum of pachymic acid (**1**). Figure S3. The UPLC chromatogram of dehydropachymic acid (**2**). Figure S4. The ESI-MS spectrum of dehydropachymic acid (**2**). Figure S5. The UPLC chromatogram of tumulosic acid (**3**). Figure S6. The ESI-MS spectrum of tumulosic acid (**3**). Figure S7. The UPLC chromatogram of dehydrotumulosic acid (**4**). Figure S8. The ESI-MS spectrum of dehydrotumulosic acid (**4**). Figure S9. The UPLC spectrum of polyporenic acid C (**5**). Figure S10. The ESI-MS spectrum of polyporenic acid C (**5**). Figure S11. The UPLC spectrum of 3-Epi-dehydrotumulosic acid (**6**). Figure S12. The ESI-MS spectrum of 3-Epi-dehydrotumulosic acid (**6**).
