# Supplementary material for: Poria cocos Lanostane Triterpenoids Extract Promotes Collagen and Hyaluronic Acid Production in D-Galactose-Induced Aging Rats
Source: Life (Basel). 2023 Oct 28;13(11):2130. doi: 10.3390/life13112130 (PMC10672192; doi:10.3390/life13112130)
Supplement: Supplementary file 1 [file life-13-02130-s001.zip › life-2632217-supplementary.pdf]

Supporting Information

Poria cocos lanostane triterpenoids extract Promote Collagen and Hyaluronic acid Production in D-Galactose-induced Aging Rats

Chien-Liang Chao[1], Han-Peng Kuo[2], Hsin-Wen Huang[1], Maw-Yeun Cheng[1], Hsin-Fan Chao[1], Shih-Min Lu[1], Hang-Ching Lin[1, 3], Chao-Jih Wang[4], Tsu-Chung Chang[5,*] and Chi-Rei Wu[6,*]

[1] Sinphar Pharmaceutical Co., Ltd., Sinphar group, Yilan 269, Taiwan;

[2] SynCore Biotechnology Co., Ltd., Sinphar group, Yilan 269, Taiwan;

[3] School of Pharmacy, National Defense Medical Center, Taipei 114, Taiwan;

[4] Sinphar Tian-Li Pharmaceutical Co., Ltd., Sinphar Group, Hangzhou 311100,

[5] Department of Biochemistry, National Defense Medical Center, Taipei 114, Taiwan;

[6] China Department of Chinese Pharmaceutical Sciences and Chinese Medicine Resources, China Medical University, Taichung 404, Taiwan.

1. The characteristics of Triterpenoid Compounds (1-6).

Table S1. ^{1}H NMR (400 MHz) and ^{13}C (100 MHz) Spectroscopic Data for **1-6**.

2. Chromatogram of Triterpenoid Compounds (1-6) using UPLC/MS.

Figure S1. The UPLC chromatogram of pachymic acid (**1**).

Figure S2. The ESI-MS spectrum of pachymic acid (**1**).

Figure S3. The UPLC chromatogram of dehydropachymic acid (**2**).

Figure S4. The ESI-MS spectrum of dehydropachymic acid (**2**).

Figure S5. The UPLC chromatogram of tumulosic acid (**3**).

Figure S6. The ESI-MS spectrum of tumulosic acid (**3**).

Figure S7. The UPLC chromatogram of dehydrotumulosic acid (**4**).

Figure S8. The ESI-MS spectrum of dehydrotumulosic acid (**4**).

Figure S9. The UPLC spectrum of polyporenic acid C (**5**).

Figure S10. The ESI-MS spectrum of polyporenic acid C (**5**).

Figure S11. The UPLC spectrum of 3-Epi-dehydrotumulosic acid (**6**).

Figure S12. The ESI-MS spectrum of 3-Epi-dehydrotumulosic acid (**6**).

1. The characteristics of Triterpenoid Compounds (1-6).

Pachymic Acid (**1**): white powder; ESI−MS m/z: 527.3822 $[M-H]^-$; ^1H NMR (400 MHz, Pyridine-d_5) and ^{13}C-NMR (100 MHz, Pyridine-d_5) data see Table S1.

Dehydropachymic Acid (**2**): white powder; ESI−MS m/z: 525.3657 $[M-H]^-$; ^1H NMR (400 MHz, Pyridine-d_5) and ^{13}C-NMR (100 MHz, Pyridine-d_5) data see Table S1.

Tumulosic Acid (**3**): white powder; ESI−MS m/z: 485.3635 $[M-H]^-$; ^1H NMR (400 MHz, Pyridine-d_5) and ^{13}C-NMR (100 MHz, Pyridine-d_5) data see Table S1.

Dehydrotumulosic Acid (**4**): white powder; ESI−MS m/z: 483.3475 $[M-H]^-$; ^1H NMR (400 MHz, Pyridine-d_5) and ^{13}C-NMR (100 MHz, Pyridine-d_5) data see Table S1.

Polyporenic Acid C (**5**): white powder; ESI−MS m/z: 481.3332 $[M-H]^-$; ^1H NMR (400 MHz, Pyridine-d_5) and ^{13}C-NMR (100 MHz, Pyridine-d_5) data see Table S1.

3-Epi-Dehydrotumulosic Acid (**6**): white powder; ESI−MS m/z: 483.3533 $[M-H]^-$; ^1H NMR (400 MHz, Pyridine-d_5) and ^{13}C-NMR (100 MHz, Pyridine-d_5) data see Table S1.

Table S1. ^{1}H NMR (400 MHz) and ^{13}C (100 MHz) Spectroscopic Data for **1**-**6**.

	**3**			**5**			**6**			**4**	
no.	δ_H multi (J in Hz)	δ_C		δ_H multi (J in Hz)	δ_C		δ_H multi (J in Hz)	δ_C		δ_H multi (J in Hz)	δ_C
1	1.65 (br t, J=13)	36.58 t		1.65 (td, J=14, 4.3); 2.09 (m)	36.8 t		2.26 (m); 1.72(br d, J=12.4)	30.6 t		1.97 (m)； 2.57 (m)	36.74 t
2	1.88 (m)；1.96 (m)	29.13 t		2.3; 2.75 (m)	34.9 t		1.88 (dd, J=13.7, 8.4) 2.07 (m)	26.7 t		1.51 (m)； 1.95 (m)	29.14 t
3	3.43 (t, J=8.0)	78.53 d			215.2 s		3.62 (s)	75.1 d		3.48 (t, J=8.0)	78.47 d
4		40.02 s			47.5 s			38.0 s			39.82 s
5	1.18 (d, J=3.8)	51.42 d		1.59 (dd, J=11.5, 3.7)	51.0 d		2.01 (br t, J=8.3)	43.7 d		1.32 (d, J=3.8)	50.27 d
6	1.56 (m)；1.76 (m)	19.21 t		1.96 (m); 2.13 (m)	23.9 t		2.10 (m); 2.10 (m)	23.4 t		2.16 (m)	23.99 t
7	2.13 (m)	27.47 t		5.57 (br d, J=6)	120.7 d		5.62 (s)	121.3 d		5.65 (br s)	121.76 d
8		135.39 s			142.8 s			142.8 s			143.19 s
9		135.29 s			144.7 s			146.7 s			146.86 s
10		37.88 s			37.5 s			37.9 s			38.34 s
11	2.01 (m)	21.46 t		5.35 (br d, J=5.6)	117.7 d		5.46 (d, J=5.7)	116.2 d		5.40 (br s)	117.02 d
12	2.20 (d, J=12.5)；2.00 (m)	30.20 t		2.37; 2.67 (br d, J=18.2)	36.3 t		2.64 (br d, J=15.6); 2.40 (m)	36.3 t		2.71 (d, J=12.5)； 2.42 (m)	36.79 t
13		49.22 s			45.0 s			45.1 s			45.56 s
14		46.76 s			49.4 s			49.5 s			49.89 s
15	1.74 (d, J=13)；2.42 (dd, J=13, 8)	44.18 t		2.42; 1.91 (d, J=13)	44.4 t		2.40 (m)	44.5 t		1.74 (d, J=13)； 2.42 (dd, J=13, 8)	44.92 t
16	4.56 (t, J=6.8)	77.16 d		4.52 (t, J=7, 6.8)	76.4 d		4.51 (t, J=7.2, 6.5)	76.5 d		4.56 (t, J=6.8)	76.91 d
17	2.80 (dd, J=11.1,	57.79 d		2.86 (dd, J=11.2,	57.6 d		2.85 (dd, J=11.1,	57.6 d		2.92 (dd, J=11.1,	58.12 d

	5.9)			5.8)			5.7)			5.9)	
18	1.17 (s)	18.28 q		1.04 (s)	17.6 q		1.08 (s)	17.7 q		1.09 (s)	18.11 q
19	1.05 (s)	19.42 q		1.12 (s)	22.0 q		1.09 (s)	23.0 q		1.09 (s)	23.47 q
20	2.93 (m)	49.28 d		2.94 (m)	48.5 d		2.94 (br t, J=9.5)	48.6 d		2.92 (m)	49.03 d
21		179.49 s			178.7 s			178.7 s			177.87 s
22	2.66 (m)；2.50 (m)	32.08 t		2.46 (m); 2.62 (m)	31.4 t		2.45 (m); 2.70 (m)	31.5 t		2.51 (m)； 2.43 (m)	31.94 t
23	2.41 (br t, J=12)；2.57 (br t, J=12)	33.72 t		2.41 (m); 2.53 (m)	33.2 t		2.37; 2.52 (t, J=11.6)	33.2 t		2.41 (br t, J=12)；2.53 (br t, J=12)	33.69 t
24		156.58 s			156.0 s			156.1 s			156.52 s
25	2.30 (m)	34.61 d		2.25 (m)	34.1 d		2.26 (m)	34.1 d		2.33 (m)	34.58 d
26	0.97 (d, J=6.75)	22.52 q		0.97 (dd, J=7.2)	22.0 q		0.96 (d, J=7)	22.0 q		1.01 (d, J=6.75)	22.48 q
27	0.98 (d, J=6.75)	22.38 q		0.99 (dd, J=7.2)	21.9 q		0.98 (d, J=7)	21.9 q		1.01 (d, J=6.75)	22.34 q
28	1.23 (s)	29. 15 q		1.12 (s)	25.7 q		1.18 (s)	29.2 q		1.23 (s)	29.31 q
29	1.06 (s)	16.87 q		1.04 (s)	22.4 q		0.98 (s)	23.2 q		1.15 (s)	17.12 q
30	1.47 (s)	25.94 q		1.44 (s)	26.4 q		1.42 (s)	26.6 q		1.53 (s)	27.08 q
31	4.83 (s)；4.97 (s)	107.01 t		4.83 (s)；4.97 (s)	107.5 t		4.83 (s); 5.02 (s)	107.1 t		4.86 (s)； 5.00 (s)	107.51 t

	**1**			**2**	
no.	δ_H multi (J in Hz)	δ_C		δ_H multi (J in Hz)	δ_C
1	1.11 (m)<br>1.60 (br t, J=13)	35.3 t		1.42 (m); 1.89 (m)	35.5 t
2	1.60 (m)<br>1.69 (m)	24.5 t		1.77(m); 1.68 (m)	24.5 t
3	4.66 (dd, J=11.6, 4.4)	80.6 d		4.70 (dd, J=12, 4)	80.6 d
4		38.0 s			37.8 s
5	1.14 (m)	50.7 d		1.25 (dd)	49.5 d
6	1.52 (m)	18.4 t		2.05 (m); 2.05 (m)	23.0 t
7	2.10 (m); 2.10 (m)	26.7 t		5.55 (br, s)	120.7 d
8		135.0 s			142.7 s
9		134.4 s			145.7 s
10		37.1 s			37.5 s
11	1.92 (m); 1.90 (m)	20.9 t		5.28 (d, J=5.6)	116.9 d
12	2.26 (t, J=6.7); 2.00 (m)	29.6 t		2.68; 2.42 (br d, J=18)	36.2 t
13		48.8 s			45.0 s
14		46.2 s			49.3 s
15	1.69 (d, J=13)<br>2.37 (dd, J=13, 9)	43.6 t		2.42; 1.92 (d, J=13)	44.3 t
16	4.51 (t, J=6.8, 6.4)	77.6 d		4.50 (t, J=8, 6.8)	76.4 d
17	2.80 (dd, J=10.8,	57.3 d		2.87 (dd, J=11.2,	57.5 d

	6.4)			5.8)	
18	1.12 (s)	17.8 q		1.02 (s)	17.6 q
19	0.94 (s)	19.2 q		0.98 (s)	21.0 q
20	2.93 (m)	48.8 d		2.92 (m)	48.5 d
21		178.8 s			178.7 s
22	2.63(m)<br>2.47 (m)	31.6 t		2.51 (m); 2.64 (m)	31.4 t
23	2.40 (br t, J=12)<br>2.53 (br t, J=12)	33.2 t		2.40 (m); 2.54 (m)	33.2 t
24		156.1 s			156.0 s
25	2.27 (m)	34.1 d		2.28 (m)	34.0 d
26	0.97 (d, J=6.8)	22.0 q		0.96 (dd, J=7)	22.0 q
27	0.99 (d, J=6.8)	21.9 q		0.97 (dd, J=7)	21.8 q
28	0.89 (s)	28.0 q		0.86 (s)	28.1 q
29	0.91 (s)	16.8 q		0.96 (s)	17.1 q
30	1.47 (s)	25.4 q		1.46 (s)	26.5 q
31	4.83 (s)<br>4.96 (s)	107.0 t		4.82 (s)<br>4.96 (s)	107.0 t

2. Chromatogram of Triterpenoid Compounds (1-6) using UPLC/MS.

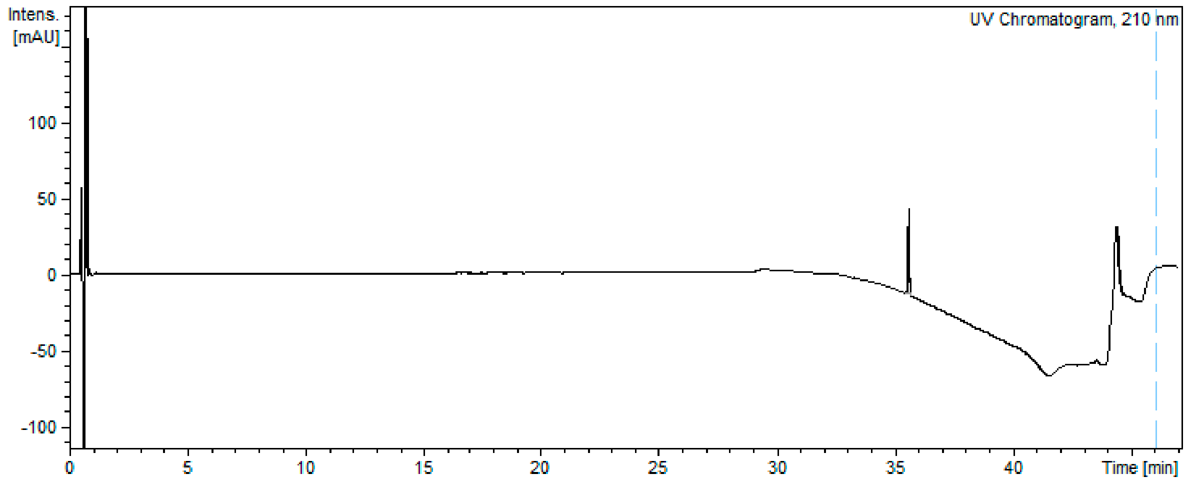

Figure S1. The UPLC chromatogram of pachymic acid (**1**).

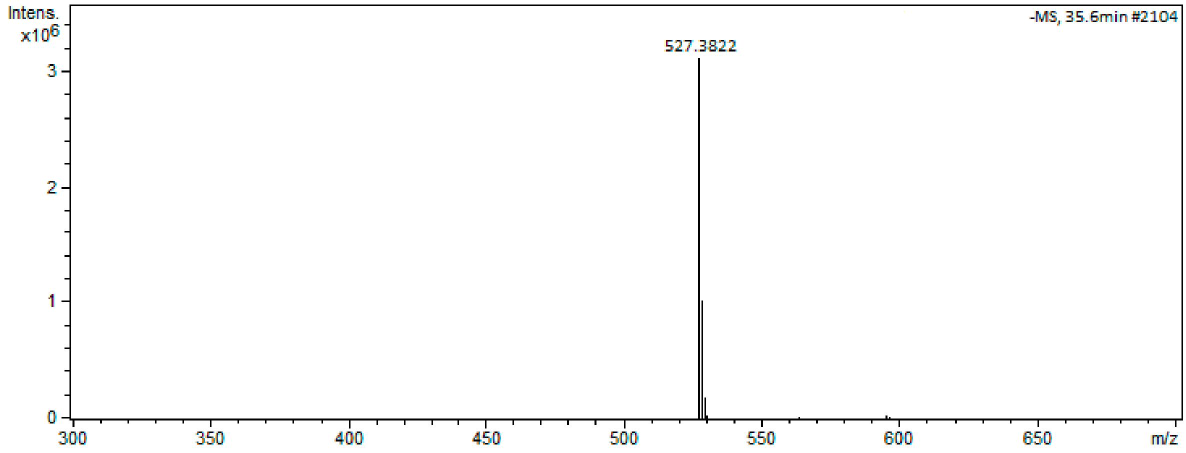

Figure S2. The ESI-MS spectrum of pachymic acid (**1**).

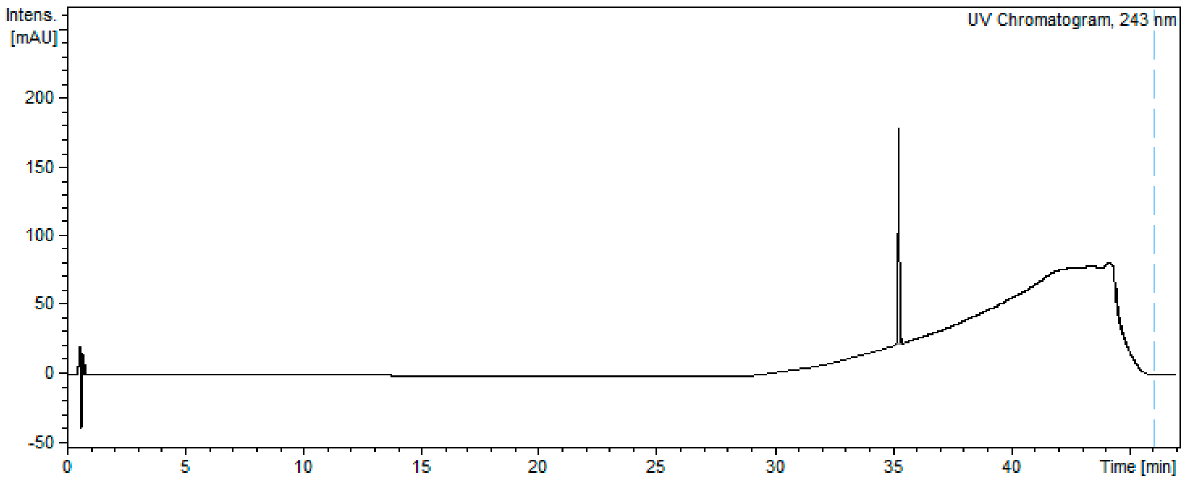

Figure S3. The UPLC chromatogram of dehydropachymic acid (2).

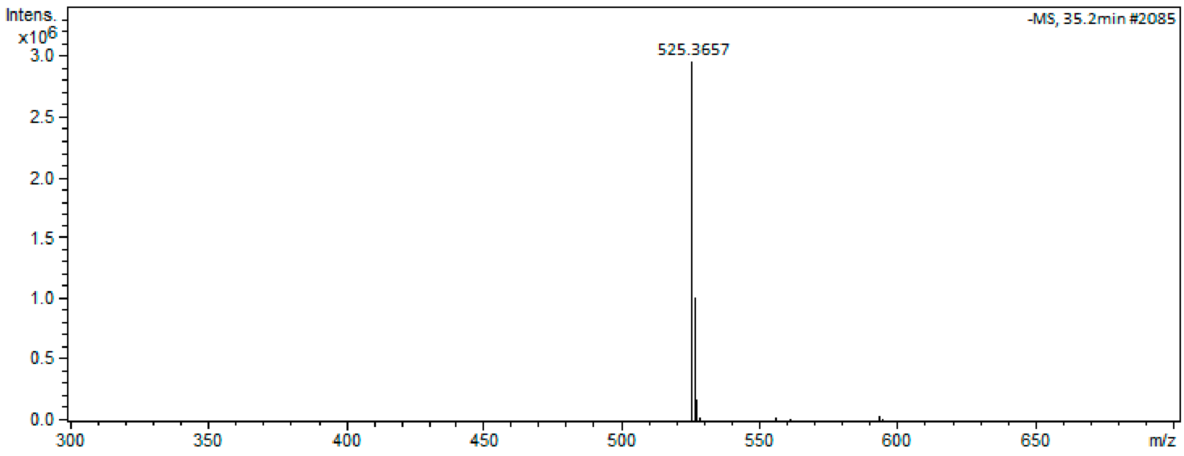

Figure S4. The ESI-MS spectrum of dehydropachymic acid (**2**).

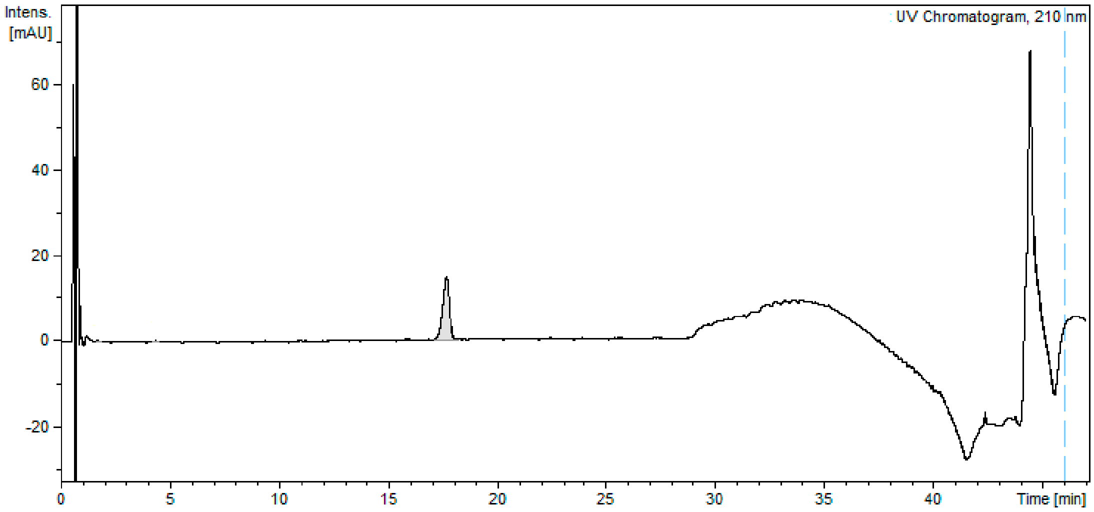

Figure S5. The UPLC chromatogram of tumulosic acid (**3**).

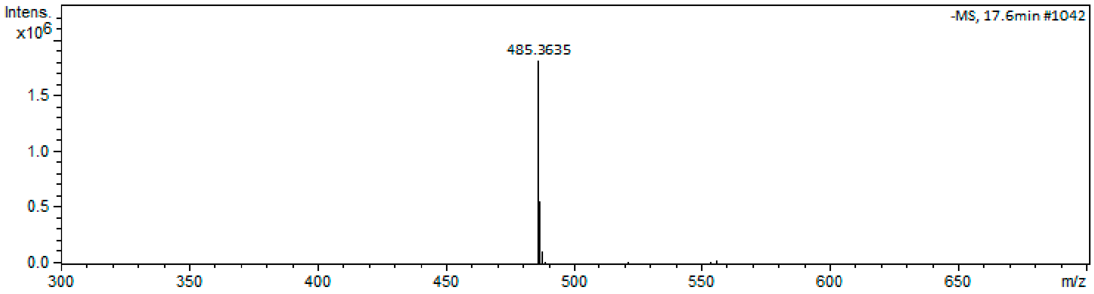

Figure S6. The ESI-MS spectrum of tumulosic acid (**3**).

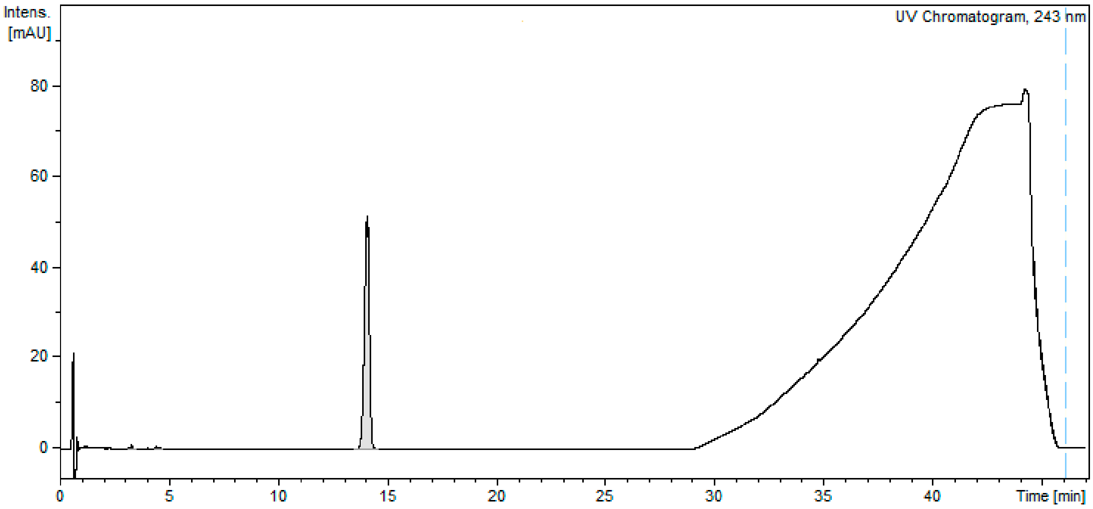

Figure S7. The UPLC chromatogram of dehydrotumulosic acid (**4**).

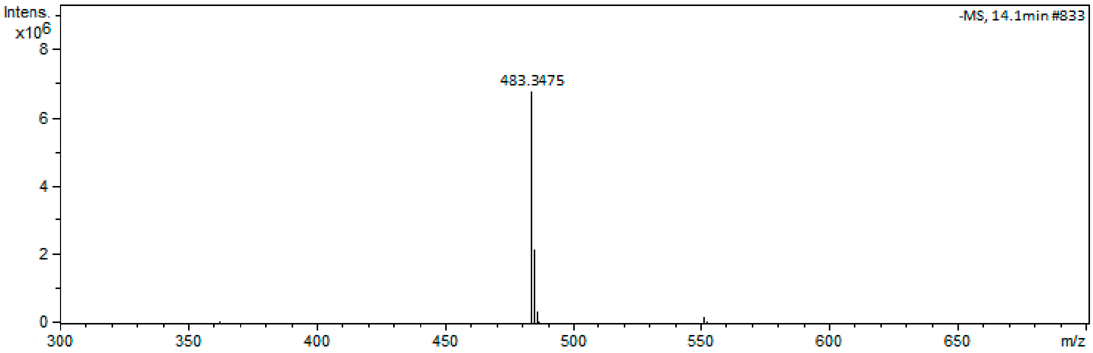

Figure S8. The ESI-MS spectrum of dehydrotumulosic acid (**4**).

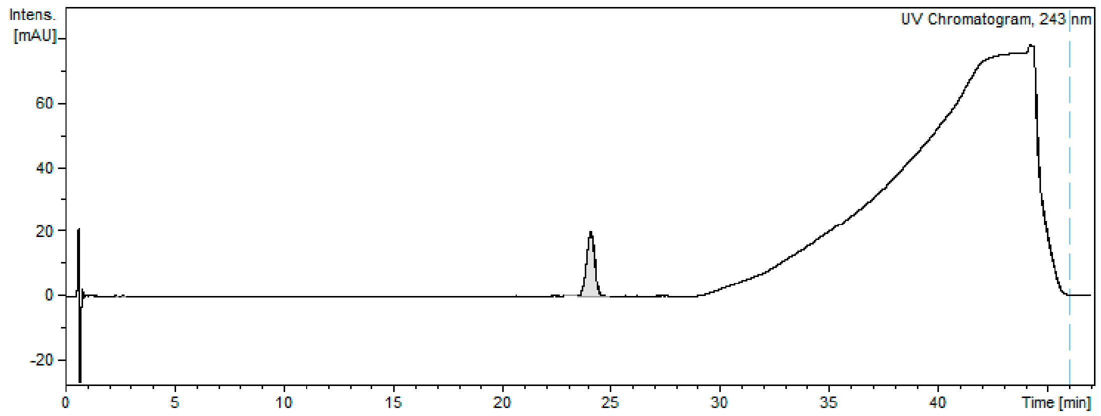

Figure S9. The UPLC chromatogram of polyporenic acid C (**5**).

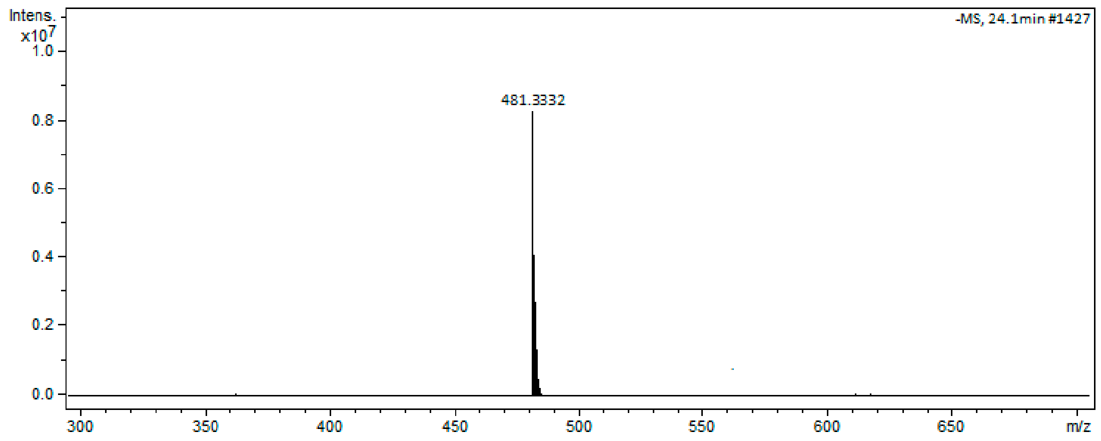

Figure S10. The ESI-MS spectrum of polyporenic acid C (**5**).

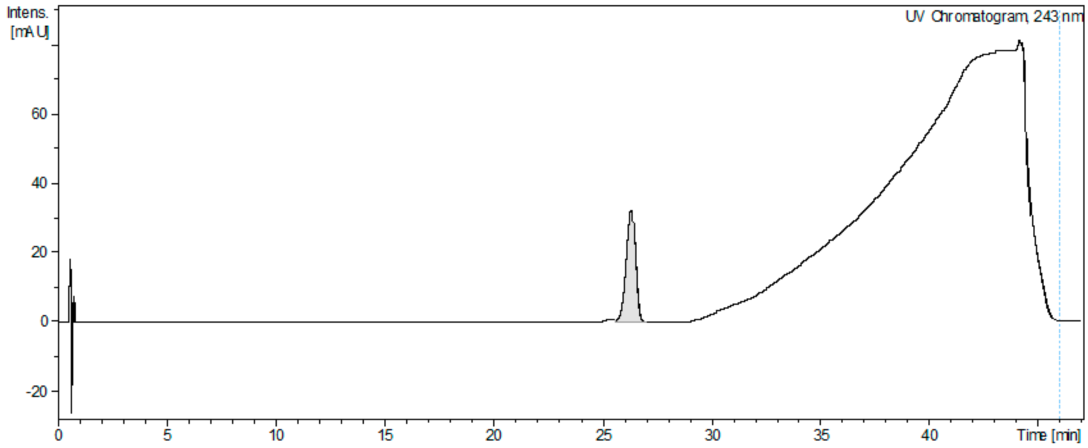

Figure S11. The UPLC chromatogram of 3-epi-dehydrotumulosic acid (**6**).

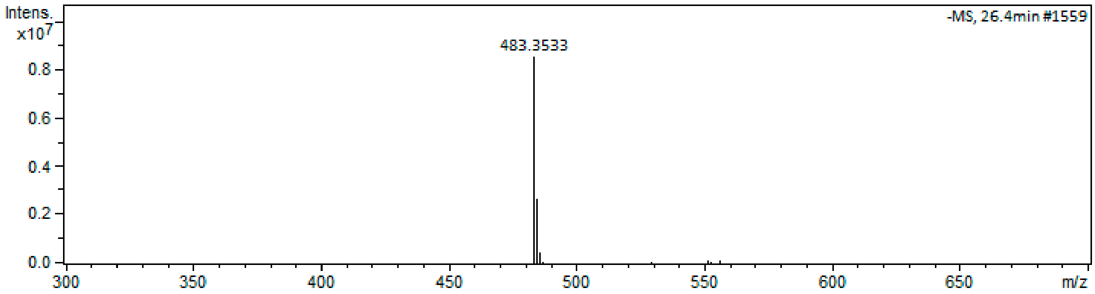

Figure S12. The ESI-MS spectrum of 3-epi-dehydrotumulosic acid (**6**).
